# Inhibition of Androgen Signalling Improves the Outcomes of Therapies for Bladder Cancer: Results from a Systematic Review of Preclinical and Clinical Evidence and Meta-Analysis of Clinical Studies

**DOI:** 10.3390/diagnostics11020351

**Published:** 2021-02-20

**Authors:** Massimiliano Creta, Giuseppe Celentano, Luigi Napolitano, Roberto La Rocca, Marco Capece, Gianluigi Califano, Claudia Collà Ruvolo, Francesco Mangiapia, Simone Morra, Carmine Turco, Lorenzo Spirito, Ferdinando Fusco, Ciro Imbimbo, Vincenzo Mirone, Nicola Longo

**Affiliations:** 1 Department of Neurosciences, Science of Reproduction and Odontostomatology, University of Naples Federico II, 80131 Naples, Italy; massimiliano.creta@unina.it (M.C.); giuseppe.celentano2@unina.it (G.C.); luigi.napolitano12@studenti.unina.it (L.N.); marco.capece@unina.it (M.C.); Gianluigi.califano@unina.it (G.C.); claudia.collaruvolo@unina.it (C.C.R.); Francesco.mangiapia@unina.it (F.M.); simone.morra@unina.it (S.M.); carmine.turco2@unina.it (C.T.); Lorenzo.spirito@unina.it (L.S.); ciro.imbimbo@unina.it (C.I.); mirone@unina.it (V.M.); nicola.longo@unina.it (N.L.); 2 Department of Woman, Child and General and Specialized Surgery, Urology Unit, University of Campania ‘Luigi Vanvitelli’, 80131 Naples, Italy; ferdinando.fusco@unicampania.it

**Keywords:** 5 alpha reductase inhibitors, androgen deprivation therapy, androgen receptor, bladder cancer

## Abstract

Bladder cancer (BCa) is an endocrine-related tumour and the activation of androgen signalling pathways may promote bladder tumorigenesis. We summarized the available preclinical and clinical evidence on the implications of the manipulation of androgen signalling pathways on the outcomes of BCa therapies. A systematic review was performed in December 2020. We included papers that met the following criteria: original preclinical and clinical research; evaluating the impact of androgen signalling modulation on the outcomes of BCa therapies. Six preclinical and eight clinical studies were identified. The preclinical evidence demonstrates that the modulation of androgen receptor-related pathways has the potential to interfere with the activity of the Bacillus Calmette Guerin, doxorubicin, cisplatin, gemcitabine, and radiotherapy. The relative risk of BCa recurrence after transurethral resection of the bladder tumour (TURBT) is significantly lower in patients undergoing therapy with 5 alpha reductase inhibitors (5-ARIs) or androgen deprivation therapy (ADT) (Relative risk: 0.50, 95% CI: 0.30–0.82; *p* = 0.006). Subgroup analysis in patients receiving 5-ARIs revealed a relative risk of BCa recurrence of 0.46 (95% CI: 0.22–0.95; *p* = 0.040). A significant negative association between the ratio of T1 BCa patients in treated/control groups and the relative risk of BCa recurrence was observed. Therapy with 5-ARIs may represent a potential strategy aimed at reducing BCa recurrence rate, mainly in patients with low stage disease. Further studies are needed to confirm these preliminary data.

## 1. Introduction

Bladder cancer (BCa) is the ninth most common cancer worldwide and the seventh most common in men [1]. Despite advances in therapy, the management of BCa patients remains suboptimal in both the early and advanced stages. Transurethral resection of the bladder tumour (TURBT) represents the standard surgical procedure for nonmuscle invasive BCa [2]. However, cancer recurrence and progression to muscle invasive disease has been described despite risk-reducing strategies such as intravesical chemo- and/or immunotherapy [3]. Radical cystectomy (RC) with pelvic lymph node dissection and urinary diversion represents the standard of care for patients with muscle-invasive disease and for selected patients with high-risk, nonmuscle invasive disease [4]. However, BCa relapses approximately 50% of patients following RC. External beam radiotherapy (EBRT) is supported by low-level evidence and should only be considered as a therapeutic option when the patient is unfit for RC or as part of a multimodality bladder-preserving approach. Therefore, new treatment strategies have been advocated to improve the outcomes of treatments for BCa [3]. The evidence demonstrates significant gender-related differences in patients with BCa in terms of both incidence and natural history [5]. Indeed, incidence is higher in males with a male-to-female ratio of 3.5:1, while advanced stage at the time of diagnosis and rapid progression are commonly observed in females [5]. The role of androgen signalling in BCa has been proven in preclinical and clinical settings and these findings have potential relevant therapeutic implications [6,7]. Emerging evidence also indicates that the manipulation of androgen signalling may influence BCa behaviour [8]. A previous systematic review and meta-analysis screening articles published up to April 2019 showed that BCa incidence was significantly lower in patients receiving therapy with 5-a reductase inhibitor (5-ARIs) or androgen deprivation therapy (ADT) [3]. Moreover, ADT has been reported to reduce BCa recurrence after TURBT [3]. These preliminary findings stimulated further research on the topic. Herein, we aimed to summarize the available preclinical and clinical findings in order to provide an updated overview of evidence about the impact of the modulation of androgen signalling pathways on the outcomes of therapies for BCa.

## 2. Materials and Methods

This analysis was conducted and reported according to the general guidelines recommended by the Primary Reporting Items for Systematic Reviews and Meta-analyses (PRISMA) statement [9].

### 2.1. Literature Search

The search was performed in the Medline (US National Library of Medicine, Bethesda, MD, USA), Scopus (Elsevier, Amsterdam, The Netherlands), and Web of Science Core Collection (Thomson Reuters, Toronto, ON, Canada) databases up to December 2020. The following terms were combined to capture relevant publications: “bladder cancer” AND (“transurethral resection of bladder tumour” OR “transurethral resection of bladder tumor” OR “radical cystectomy” OR “radiotherapy” OR “intravesical chemotherapy” OR “intravesical immunotherapy” OR “Bacillus Calmette-Guérin” OR “systemic chemotherapy”) AND (“androgen” OR “androgen receptor” OR “androgen deprivation therapy” OR “dutasteride” OR “finasteride” OR “castration” OR “LHRH agonist” OR “LHRH antagonists”). Reference lists in relevant articles and reviews were also screened for additional studies. 

### 2.2. Selection Criteria

Two authors (M.C. and N.L.) reviewed the records separately and individually to select relevant publications, with any discrepancies resolved by a third author (L.N.). To assess the eligibility for the systematic review, PICOS (participants, intervention, comparisons, outcomes, study type) criteria were used. PICOS criteria for preclinical studies were set as follows: (P)articipants—animal, in vitro, in vivo models of urothelial BCa; (I)ntervention—modulation of androgen signalling; (C)omparator—none; (O)utcome: evaluation of the impact of the modulation of androgen signalling on treatments used for BCa; (S)tudy types—animal, in vitro, in vivo studies. 

PICOS criteria for clinical studies were set as follows: (P)articipants—BCa patients undergoing therapies for BCa; (I)ntervention—modulation of androgen signalling (C)omparator—patients not undergoing modulation androgen signalling; (O)utcome: BCa-specific outcomes; (S)tudy types—prospective and retrospective studies, case series.

### 2.3. Data Collection

The following data were extracted from preclinical studies: first author, year of publication, study design, study model, type of androgen signalling pathway modulation, study groups, type of BCa treatment, findings, involved pathway. 

The following data were extracted from clinical studies: first author, study design, BCa stage and grade, patients’ gender and age, type and duration of androgen signalling modulation, comparators, type of BCa treatment, follow-up duration, outcome assessed. Risk of bias for clinical studies was assessed using the Risk of Bias in Nonrandomized Studies of Interventions (ROBINS-I) tool [10]. 

### 2.4. Statistical Analysis

The meta-analysis was performed by ProMeta 3 software when there were two or more studies reporting the same outcome under the same definition. The effect size (ES) was estimated by Risk Ratio (RR) reported with its 95% confidence interval (CI). Heterogeneity among studies was evaluated using the I^2^ statistics. A *p* <0.05 was considered statistically significant. To calculate the pooled effect, a random effect model was applied. Egger’s linear regression test and Begg and Mazumdar’s rank correlation test were also used to evaluate the publication bias of studies included in the meta-analysis. A metaregression analysis was used to assess the relationship between the ratio of patients with T1 BCa among those treated with 5-ARIs/controls and the RR of tumour recurrence. Subgroup analyses were performed according to the strategy of androgen signalling manipulation.

## 3. Results

**Figure 1:**
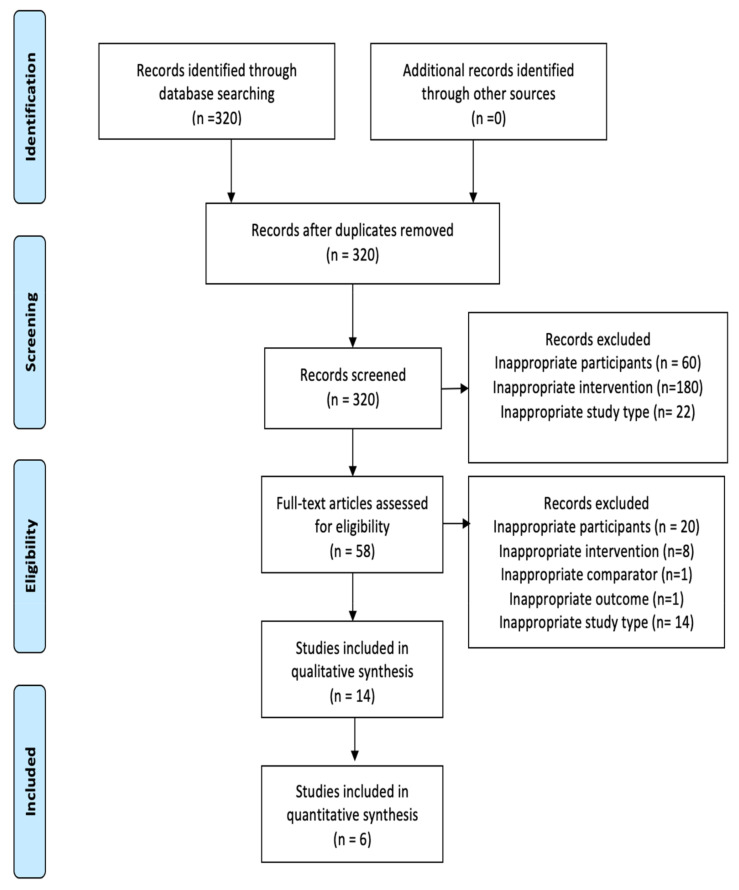
Flow diagram of the systematic review.

The search strategy revealed a total of 320 results. The screening of the titles and the abstracts defined 58 papers eligible for inclusion. Further assessment of eligibility, based on the study of the full-text papers, led to the exclusion of 44 papers. Finally, 14 studies (6 preclinical and 8 clinical) were included in the final analysis (Figure 1) [11,12,13,14,15,16,17,18,19,20,21,22,23,24]. Of these, 6 studies were included in the quantitative synthesis. 

### 3.1. Evidence from Preclinical Studies

Overall, six preclinical studies were identified investigating the effects of the manipulation of androgen signalling on the outcomes of therapies for BCa [11,12,13,14,15,16] (Table 1). 

Two studies investigated the effects of the inhibition of androgen signalling on the interaction between BCG and BCa cell lines in in vitro and in vivo models [11,13]. The authors showed an increased amount of intracellular BCG, increased rate of BCG positive cells, increased BCG-related cytotoxic activity, increased recruitment of monocytes/macrophages and decreased cell proliferation following the inhibition of androgen signalling in in vitro models [11,13]. The suppression of BCa cell proliferation following the inhibition of androgen signalling was also proven in an in vivo mouse model [13]. Molecular pathways involved in improved BCG efficacy following the inhibition of androgen signalling include Rab27b, interleukin 6, integrin α5β1, High Mobility Group Box 1.

One study investigated the effects of flutamide and its major active metabolite hydroxyflutamide on the radiosensitivity of BCa cells in in vivo and in vitro settings, respectively [12]. The authors demonstrated the enhanced inhibitory effects of radiation following inactivation of the androgen receptor mediated by the modulation of the ATR, CHEK1 and PARP-1 pathways [12]. 

One study investigated the effects of androgen receptor blockage on BCa cell lines exposed to doxorubicin by showing increased drug activity [14]. 

One study investigated the impact of androgen receptor activation on cisplatin sensitivity by demonstrating reduced cisplatin sensitivity following exposure to the synthetic androgen R1881 [15]. 

One study investigated the effects of enzalutamide on cell growth following exposure to gemcitabile and found decreased cell viability following exposure to enzalutamide [16]. 

### 3.2. Evidence from Clinical Studies

Overall, eight retrospective case-control studies were identified involving a total of 17,454 male patients [17,18,19,20,21,22,23,24]. The study characteristics, patients’ demographics and tumour features are summarized in Table 2. The manipulation of the androgen signalling pathway involved the use of 5-ARIs in 771patients (dutasteride, finasteride, and unspecified drug in 215, 8, and 548 patients, respectively), gonadotropin-releasing hormone agonists in 10 patients, peripheral antiandrogen in 7 patients, gonadotropin-releasing hormone agonists and/or peripheral antiandrogen in 13 patients and unspecified drug in 86 patients. BCa treatment modality included TURBT (7 studies), RC (2 studies), RT (1 study). The following outcomes were assessed: recurrence-free survival, progression-free survival, cancer-specific survival, overall survival. The risk of bias evaluation with ROBINS-I indicates the presence of a moderate bias for all studies (Table 3). 

#### 3.2.1. BCa Recurrence Following TURBT

**Figure 2:**
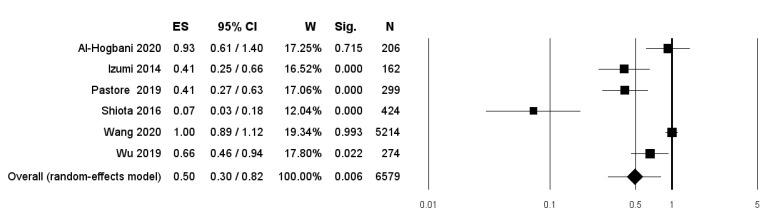
Forest plot showing the association between use of (5-ARIs) or androgen deprivation therapy (ADT) and the Risk Ratio (RR) of bladder cancer (BCa) recurrence. ES: Effect Size; CI: Confidence Interval. (I^2^ = 91.4, *p* = 0.000).

BCa recurrence after TURBT was investigated in six studies [14,15,16,17,20,21,22,23]. The overall pooled data from these studies showed a statistically significant lower RR (RR: 0.50, 95% CI: 0.30–0.82, *p* = 0.006) in patients undergoing therapy with 5-ARIs or ADT compared to controls (Figure 2). 

**Figure 3:**
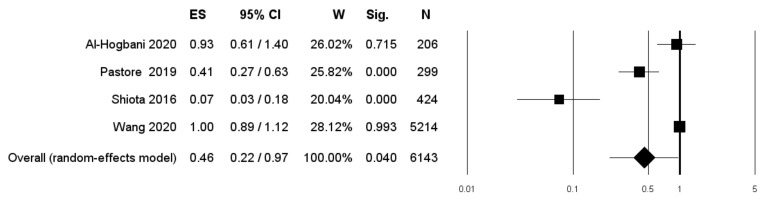
Forest plot showing the association between use of 5-ARIs and the RR of BCa recurrence. ES: Effect Size; CI: Confidence Interval. (I^2^ = 93.4, *p* = 0.000).

Four studies included only patients undergoing therapy with 5-ARIs [17,21,22,23]. Pooled data from these studies confirmed a statistically significant lower RR of BCa recurrence (RR: 0.46 (95% CI: 0.22–0.95; *p* = 0.040) (Figure 3). 

**Figure 4:**
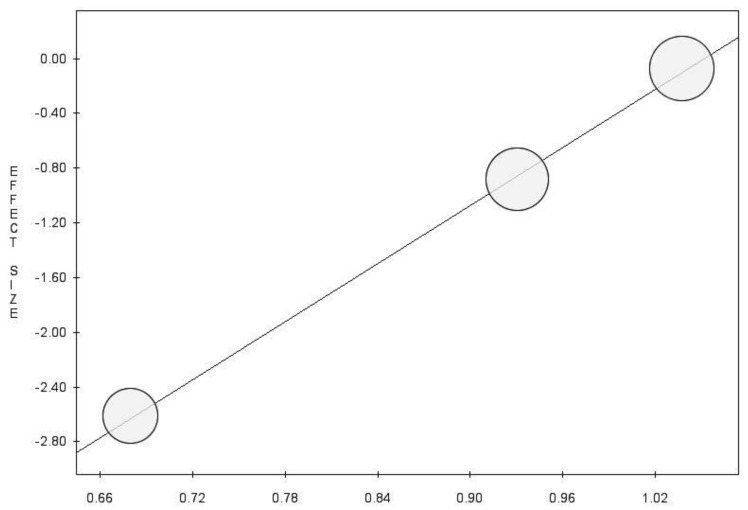
Metaregression of the proportion of patients with T1 BCa among those treated with 5-ARIs / proportion of patients with T1 BCa among those not treated with 5-ARIs and the RR of BCa recurrence on the RR of BCa recurrence (y = −7.44 + 7.07 x, *p* = 0.014).

Metaregression demonstrated a statistically significant negative correlation between the proportion of patients with T1 BCa among those treated with 5-ARIs/proportion of patients with T1 BCa among those not treated with 5-ARIs and the RR of BCa recurrence (*p*:0.014) (Figure 4). 

#### 3.2.2. BCa Progression

**Figure 5:**
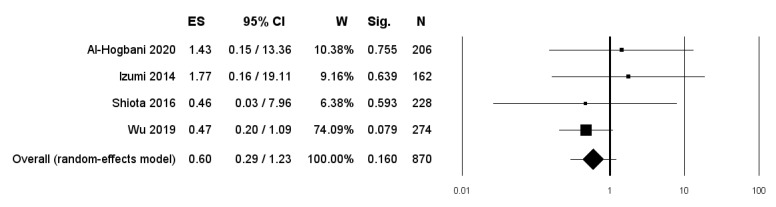
Forest plot showing the association between use of 5-ARIs or ADT and the RR of BCa progression. ES: Effect Size; CI: Confidence Interval. (I^2^ = 0.00, *p* = 0.633).

BCa progression was investigated in 4 studies [17,20,21,24]. The overall pooled data showed a nonstatistically significant lower RR in patients undergoing therapy with 5-ARIs or ADT compared to controls (RR: 0.60, 95% CI: 0.29–1.23, *p* = 0.160) (Figure 5). 

**Figure 6:**
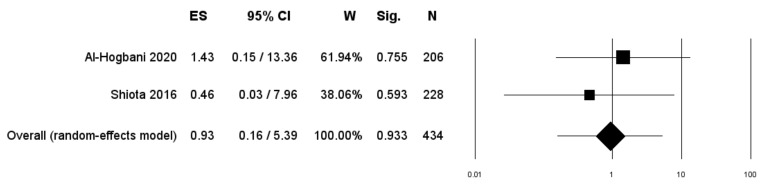
Forest plot showing the association between use of 5-ARIs and the RR of BCa progression. ES: Effect Size; CI: Confidence Interval. (I^2^ = 0.00, *p* = 0.540).

Two studies included only patients undergoing therapy with 5-ARIs [17,21]. Pooled data from these two studies revealed a RR of BCa progression of 0.93 (95% CI: 0.16–5.39; *p* = 0.933) (Figure 6). 

#### 3.2.3. BCa Recurrence Following RC

One study investigated the impact of therapy with 5-ARIs on recurrence free survival following RC [18]. The use of 5-ARIs was not an independent predictor of recurrence free survival [18].

#### 3.2.4. BCa-Related Mortality

BCa-related mortality was investigated in three studies [18,19,23]. In the study by Wang et al. the number of deaths caused by BCa was 163 (34.39%) and 1873 (39.51%) in the 5-ARIs, and in the control group, respectively [23]. Compared to nonusers, patients who received 5-ARIs showed a lower risk of BCa death in multivariable adjusted analysis (Odds Ratio: 0.835, 95% CI: 0.71–0.98) [23]. Similarly, in the study by Makela et al., patients who received 5-ARI before and after BCa diagnosis showed a lower risk of BCa death on multivariable adjusted analysis (HR: 0.85 and 0.77, respectively) [19]. McMartin et al. failed to find significant differences for 5-ARI use on cancer specific survival after RC on adjusted Cox regression analysis [18].

**Figure 7:**
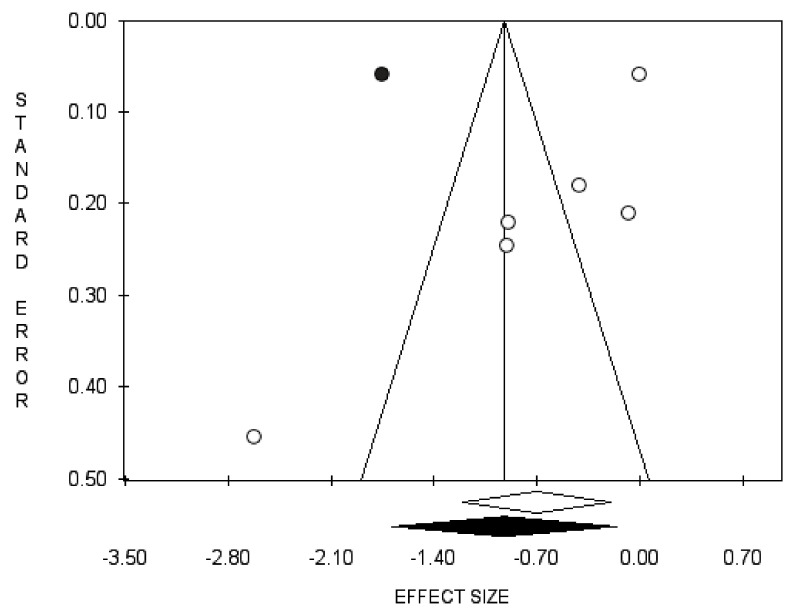
Funnel plots of the meta-analysis evaluating the association between use of 5-ARIs or ADT and the RR of BCa recurrence. Egger’s linear regression (t = −3.84, *p* = 0.019) and Begg and Mazumdar rank correlation test (z = −2.07, *p* = 0.039).

**Figure 8:**
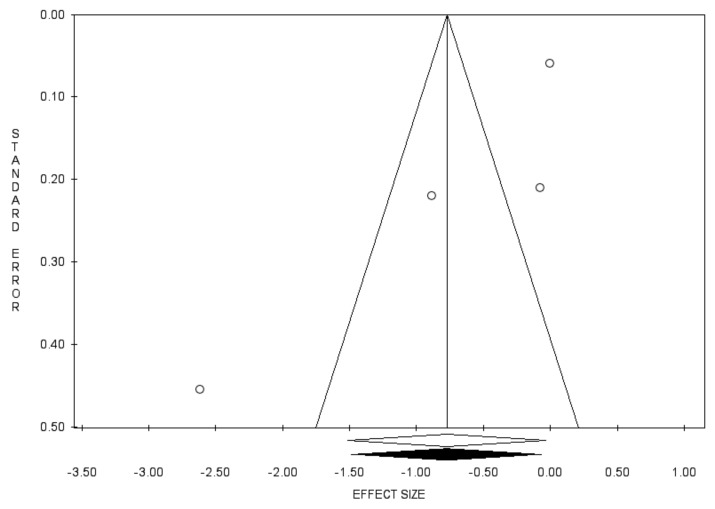
Funnel plots of the meta-analysis evaluating the association between use of 5-ARIs and the RR of BCa recurrence. Egger’s linear regression (t = −2.39, *p* = 0.139) and Begg and Mazumdar rank correlation test (z = −2.04, *p* = 0.042).

**Figure 9:**
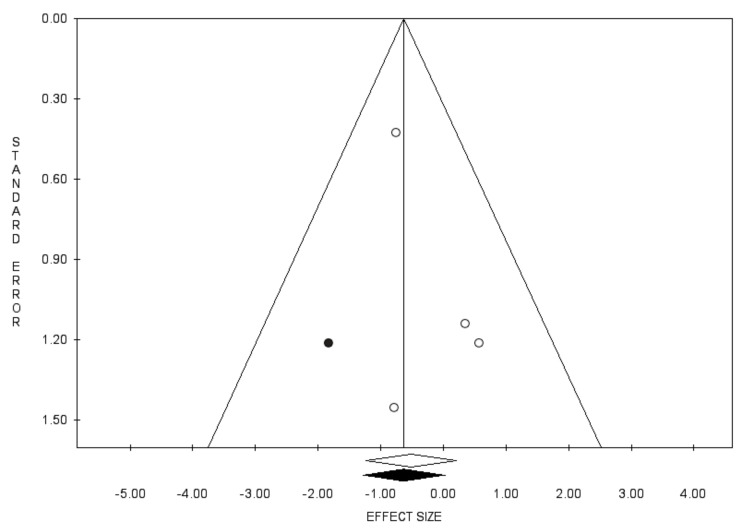
Funnel plots of the meta-analysis evaluating the association between use of 5-ARIS or ADT and the RR of BCa progression. Egger’s linear regression (t = 1.46, *p* = 0.282) and Begg and Mazumdar rank correlation test (z = 0.68, *p* = 0.497).

Bias evaluation is reported in Figure 7, Figure 8 and Figure 9. 

## 4. Discussion

The current evidence supports the hypothesis that BCa is a endocrine-related tumours and that the modulation of androgen-dependent pathways can alter tumour behaviour [25]. Indeed3 androgen signalling can promote DNA breaks and chromosomal rearrangements, leading to the formation of oncogenic fusion genes [3]. The modulation of androgen signalling pathways as complementary strategy in BCa patients represents a promising field of investigation [25,26]. The results from the present systematic review demonstrate that strategies aimed at suppressing androgen signalling can improve the outcomes of therapies for BCa in both preclinical and clinical settings. The available preclinical evidence from both in vitro and in vivo models demonstrate that the manipulation of androgen-dependent pathways can interfere with the sensitivity of BCa to BCG, radiation, and chemotherapy. In detail, the experimental negative modulation of the androgen-dependent pathway can increase the amount of intracellular BCG, the rate of BCG positive cells, BCG efficacy to suppress BCa cell proliferation, BCG cytotoxic activity, and BCG-induced recruitment of monocytes/macrophages. Moreover, the negative modulation of the androgen-dependent pathway can enhance the sensitivity of the BCa cell lines to radiation and chemotherapy in preclinical experimental settings. These findings are in line with immunohistochemical findings showing that androgen receptor expression is associated with resistance to BCG and to cisplatin [11,15].

Despite these findings, the differential impact of different subtypes of androgen suppression modalities on BCa behaviour in preclinical models remains controversial. Indeed, in their in vitro study, Nagata et al. demonstrated that, unlike androgen receptor antagonists, 5-ARIs have no significant impact on the development and progression of urothelial cancer [27]. 

The clinical evidence synthesized in the present review demonstrates a statistically significantly lower risk of BCa recurrence in the overall population of patients undergoing hormonal manipulation with ADT or 5-ARIs. One study included only patients undergoing ADT and found it to be an independent prognosticator for BCa recurrence at multivariable analysis (Hazard Ratio: 0.29; 95% CI: 0.17–0.49) [24]. The pooled analysis relative to the subgroup of patients undergoing 5-ARIs therapy confirmed a statistically significant reduction of BCa recurrence risk with respect to controls. Interestingly, we found, for the first time, a statistically significant inverse relationship between the proportion of patients with T1 disease among patients receiving 5-ARIs with respect to controls and the magnitude of the RR of BCa recurrence. 

Despite the beneficial effects in terms of BCa recurrence, the pooled data do not support a protective role by ADT and/or therapy with 5-ARIs in terms of BCa progression in the overall population of subjects evaluated. However, some subsets of patients may benefit from androgen suppression also in terms of progression. Wu et al. found that therapy with ADT or 5-ARIs was potentially associated with lower progression rates in patients with low/intermediate-risk BCa, but not in their high-risk counterparts. Indeed, the 5-yr progression free survival with and without ADT or 5-ARIs was 49% versus 55% (*p* = 0.76) in high-risk BCa and 100% versus 74% (*p* = 0.073) in low/intermediate-risk BCa [20]. Studies evaluating the impact of therapy with 5-ARIs on BCa related mortality provided conflicting results [18,19,23]. Taken together, these findings support a protective role exerted by androgen suppression therapy mainly in the early phase of the natural history of BCa. The benefits provided by androgen suppression (obtained through 5-ARIs or ADT) concern both BCa recurrence and progression. However, based on the available evidence, these advantages seem to be related to the aggressiveness of the disease. These findings should be interpreted in light of previous evidence demonstrating that, in BCa patients, androgen receptor expression inversely correlates with tumour grade and stage [28]. Therefore, it can be hypothesized that the lower expression of androgen receptors in patients with high-stage and high-grade tumours may represent the substrate for the lower efficacy of androgen suppression strategies in terms of both recurrence and progression. Accordingly, Izumi et al. found that androgen receptor expression in nonmuscle invasive BCa was a predictor of the preventive effect of ADT on tumour recurrence [29]. The lower expression of androgen receptors in patients with high stage and/or high grade BCa may also be responsible, in part, for the apparent discrepancy between preclinical evidence obtained in cell lines in vitro demonstrating higher efficacy of BCG (a drug mainly prescribed to prevent BCa progression) following suppression of androgen signalling and clinical evidence that do not confirm the protective role of androgen suppression in terms of BCa progression in the overall population. Unfortunately, there are no clinical studies about the efficacy of androgen suppression on the outcomes of chemo/radiotherapy. The results from the present systematic review have relevant clinical implications and pose the basis for further investigations ADT is widely adopted in patients with metastatic disease and 5-ARIs have been evaluated extensively for the primary prevention of prostate cancer [30]. The potential benefit of 5-ARIs and ADT for BCa patients is a recent finding. Based on this data, we can hypothesize a synergistic effect by androgen suppression strategies and standard treatments in early stage BCa in male patients. However, further studies are needed to confirm these observations. Indeed, given the concomitant bladder outflow benefits and a favourable safety profile, there is a compelling rationale for the prospective evaluation of 5-ARIs as a secondary prevention strategy for BCa recurrence in men [31,32,33]. Although androgen suppression has the potential of being a means of radio- and chemiosensitization, further studies are needed to confirm this preclinical evidence. 

The potential limits of the available literature must be acknowledged: the available studies are few, often of low methodological quality, and with short follow-up. Moreover, they enrol only male patients with heterogeneous BCa features and only a small percentage of studies focused on patients undergoing RC or EBRT. Further studies are needed to better investigate the role of androgen suppression in specific subgroups of BCa patients (i.e., young versus older patients), to compare the effects of 5-ARIs to ADT, and to better elucidate the impact of androgen manipulation strategies in patients with muscle invasive disease undergoing RC or EBRT. 

## 5. Conclusions

The suppression of androgen signalling improves BCa cell chemosensitivity and radiosensitivity, as well as BCG efficacy in the preclinical setting. The clinical evidence demonstrates that the RR of BCa recurrence after TURBT is significantly lower in patients undergoing androgen suppression therapy with 5-ARIs/ADT compared to controls and that the magnitude of this protective effect is inversely related to tumour stage. Despite the existence of a trend toward the benefits of androgen suppression in terms of BCa progression in patients with low-intermediate risk, these findings deserve further investigations.

## Figures and Tables

**Table 1:** Summary of preclinical studies.

Study	Study Design	Model	Androgen-Related Pathway Manipulation	BCa Treatment	Outcome Assessed	Findings	Involved Pathway
Mizushima [11]	In vitro	Cell Lines (UMUC3, TCCSUP, and 5637-AR)	AR knockdown AR overexpression	BCG	Amount of intracellular BCG	↑ amount of intracellular BCG in AR knockdown cell lines, ↓ amount of intracellular BCG in AR-positive cells, ↓ amount of intracellular BCG in AR overexpression cells	Rab27b
					Rate of BCG positive cells	↑ rate of BCG positive cells in AR knockdown cell lines, ↓ rate of BCG positive cells in AR-positive cells, ↓ rate of BCG positive cells in AR overexpression cells	
					BCG activity	↑ BCG activity in AR knockdown cell lines, ↓ in AR-positive cells, ↓ BCG activity in AR overexpression cells	
Ide [12]	In vitro	Cell lines (UMUC3, 5637-AR, and 647V-AR)	HF DHT, AR overexpression, and AR knockdown	RT	Radiosensitivity	↑ radiosensitivity following AR inactivation, less significant decrease of cell viability following exposure to DHT, delay of doublestrand break repair following AR knockdown	ATR, CHEK1, and PARP-1
	In vivo	Mouse xenograft	Flutamide			↑ inhibitory effects of irradiation following AR inactivation	n/a
Shang [13]	In vitro	Cell line (253J)	AR degradation enhancer ASC-J9HF	BCG	BCG attachment/internalization	↑ BCG attachment/internalization to BCa cells following exposure to HF and ASC-J9®	Integrin α5β1
					Monocytes/macrophages recruitment	↑ recruitment of monocytes/macrophages following exposure to HF and ASC-J9®	IL6 expression
					BCG activity	↑ BCG intra-cellular efficacy to suppress BCa cell proliferation following exposure to HF or ASC-J9®	HMGB1 release
	In vivo	BBN- induced BCa mouse model	ASC-J9		BCG activity	↑ BCG efficacy to suppress BCa cell proliferation following exposure to ASCJ9®	n/a
Shiota [14]	In vitro	Cell lines (UMUC3 and MBT-2)	Bicalutamide and Flutamide	CT	Doxorubicin activity	↑ doxorubicin activity following exposure to AR antagonists	n/a
Kashiwagi [15]	In vitro	Cell lines (UMUC3, 647V-AR, 5637-AR)	Synthetic androgen R1881, HF, AR overexpression, and AR knockdown	CT	Cisplatin activity	↓ cisplatin activity following exposure to R1881, ↑ cisplatin activity following exposure to HF	NF-κB
Kameyama [16]	In vitro	Gemcitabine-resistant T24 cells	Enzalutamide	CT	Gemcitabine activity	↑ gemcitabile ctivity following exposure to enzalutamide	Cyclin D1 expression

253J: human BCa cell line 253J; 647-V: human urothelial bladder carcinoma; 5637: Human bladder carcinoma cell line; AR: Androgen Receptor; BBN: N-butyl-N-(4-hydroxybutyl) nitrosamine; BCG: Bacillus Calmette Guerin; CT: Chemotherapy; DHT: dihydrotestostrone; HF: Hydroxyflutamide; HMGB1: High Mobility Group Box 1; IL: Interleukin; MBT-2: Mouse Bladder Tumor line-2; RT: Radiotherapy; TCCSUP: Human urinary bladder transitional cell carcinoma; UMUC3: Human Bladder Transitional Cell Carcinoma; ↑: increase; ↓: reduction.

**Table 2:** Summary of clinical studies.

Study	Sample Size (n)	BCa Stage, (n)	BCa Grade		Age, Years, Mean (Range)	Androgen Dependent Signalling Pathway Modulation		Exposure (n) vs Comparator (n)	BCa Treatment Type (n)	Adjuvant Intravesical Chemotherapy (n)	FU (y)	Outcome	Findings	Risk Estimate
			Low (n)	High (n)		Type (n)	Duration (months)							
Pastore [22]	312	pTa (117) pT1 (165) ≥pT2 (30)	92	220	75.1 (n/a)	Dutasteride (165)	≥12	5-ARI (165) vs no 5-ARI (147)	TURBT (312)	MMC (129) BCG (153)	2.6	RFS	↑ in 5-ARI users	HR: 0.64 (95% CI: 0.58–0.87) *p* = 0.006
												PR	No significant difference	RR: 1.43 (95% CI: 0.15-13.36) *p* = 0.755
Al-Hogbani [17]	206	pTa (96) pT1 (82) n/a (28)	29	177	70.0(68–71)	Finasteride (8) Dutasteride (31)	>6	5-ARI (39) vs no 5-ARI (167)	TURBT (206)	BCG (206)	3.3	RFS	↑ in 5-ARI users	HR: 1.00 (95% CI: 0.55–1.79) *p* = 0.72
Wang [23]	5214	n/a	n/a	n/a	76.5 (n/a)	Finasteride (n/a) Dutasteride (n/a)	n/a	5-ARI (474) vs no 5-ARI (4740)	TURBT (5214)	n/a	n/a	MR	↓ in 5-ARI users	OR: 0.826 (95% CI: 0.7–0.97) *p* = 0.018
												RFS	No significant difference	OR: 0.960 (95% CI: 0.83–1.12) *p* = 0.109
Wu [20]	274	n/a	n/a	n/a	68.3 (n/a)	5-ARI (26) GnRH agonist (10) and/or antiandrogen (7)	19 ^§^	AST (36) vs no AST (238)	TURBT (274)	BCG (245) MMC (20) Other (5)	3.1	RFS	↑ in AST users	HR: 0.53 (95% CI: 0.30–0.88) *p* = 0.01
												PFS	No significant difference	5-yr PFS with vs without AST: 80% vs 63% *p* = 0.23
												PR	No significant difference	RR: 0.47 (95% CI: 0.20–1.09) *p* = 0.079
McMartin [18]	338	pTis (34) pTa (6) pT1 (31) pT2 (63) pT3 (136) pT4 (68)	129	209	n/a	Finasteride (n/a) Dutasteride (n/a)	n/a	5-ARI (48) vs no 5-ARI (290)	RC (338)	n/a	3.0	OS	↑ in 5-ARI users	HR for OS 0.40 (95% CI: 0.19–0.83) *p* = 0.015
												CSS	No significant difference	*p* = 0.3
												RFS	No significant difference	n/a
Makela [19]	10720	n/a	n/a	n/a	n/a	Finasteride (n/a) Dutasteride (n/a)	n/a	5-ARI (n/a) vs no 5-ARI (n/a)	TURBT (n/a) RC (n/a) EBRT (n/a) CT (n/a)	n/a	4.2	CSS	↓ in 5-ARI users	HR: 0.77 (95% CI 0.68–0.88) *p* = n/a
Shiota [21]	228	pTa (157) pT1 (50) pTis (21)	101	127	70.0 (63–78)	GnRH agonist or bicalutamide (13)Dutasteride (19)	28 ^§^	AST (32) vs no AST (196)	TURBT (228)	None/single chemotherapeutic agent (51) Continuous chemotherapeutic agent (109) BCG (68)	3.6	RFS	↑ in AST users	HR: 0.36 (95% CI: 0.11– 0.89) *p* = 0.024
												PR	No significant difference	RR: 0.46 (95% CI: 0.03– 7.96) *p* = 0.593
Izumi [24]	162	pTa (105) pT1 (49)	114	37	72.7 (54–92)	n/a	n/a	ADT (86) vs no ADT (76)	TURBT (162)	Antracyclines (38) BCG (38)	13.0	RFS	↑ in ADT users	HR 0.29 (95% CI: 0.17–0.49) *p* < 0.001
												PR	No significant difference	RR: 1.77 (95% CI: 0.16–19.11) *p* = 0.639

5-ARI: 5 α reductase inhibitor; ADT: androgen deprivation therapy; AST: androgen suppression therapy; BCa: Bladder cancer; BCG: Bacillus Calmette–Guérin; CI: Confidential interval; CSS: Cancer specific survival; FSR: free survival rate; HR: Hazard ratio; FU: Follow-up; GnRH: Gonadotropin releasing hormone; IQR: Interquartile range; MIBC: Muscular invasive bladder cancer; MMC: Mitomycin; MR: Mortality rate; OR: Odds ratio; OS: Overall survival; PFS: Progression free survival; PR: Progression rate; R: Retrospective; RFS: Recurrence free survival; TURBT: Transurethral resection of bladder; §: Median value; y: Years; RC: Radical cystectomy; EBRT: External Beam Radiotherapy; CT: Chemotherapy; ↑: increase; ↓: reduction.

**Table 3:** Summary of risk of bias among included studies using Risk of Bias In Nonrandomized Studies - of Interventions (ROBINS-I) tool.

	Al-Hogbani [17]	McMartin [18]	Mäkelä [19]	Wu [20]	Shiota [21]	Pastore [22]	Wang [23]	Izumi [24]
Bias due to confounding	M	M	M	M	M	M	M	M
Bias in selection of participants into the study	L	L	M	L	L	L	M	L
Bias in classification of interventions	M	L	L	L	L	L	L	M
Bias due to deviations from intended interventions	M	L	M	L	L	L	M	L
Bias due to missing data	L	L	n/a	S	n/a	n/a	n/a	n/a
Bias in measurement of outcomes	L	L	M	L	M	M	M	M
Bias in selection of the reported result	L	S	L	L	L	L	L	M
Overall	M	M	M	M	M	M	M	M

L: Low; M: Moderate; S: Serious; n/a: data not available.

## Data Availability

Not applicable.
